# Measurement of metabolic tumor volume: static versus dynamic FDG scans

**DOI:** 10.1186/2191-219X-1-35

**Published:** 2011-12-14

**Authors:** Patsuree Cheebsumon, Floris HP van Velden, Maqsood Yaqub, Corneline J Hoekstra, Linda M Velasquez, Wendy Hayes, Otto S Hoekstra, Adriaan A Lammertsma, Ronald Boellaard

**Affiliations:** 1 Department of Nuclear Medicine & PET Research, VU University Medical Center, P.O. Box 7057, Amsterdam, 1007 MB, The Netherlands; 2 Department of Nuclear Medicine, Jeroen Bosch Hospital, 's-Hertogenbosch, 5223 GZ, The Netherlands; 3 Bristol-Myers Squibb, Princeton, NJ, 08543, USA

**Keywords:** tumor delineation, tumor volume, FDG PET, Patlak, SUV

## Abstract

**Background:**

Metabolic tumor volume assessment using positron-emission tomography [PET] may be of interest for both target volume definition in radiotherapy and monitoring response to therapy. It has been reported, however, that metabolic volumes derived from images of metabolic rate of glucose (generated using Patlak analysis) are smaller than those derived from standardized uptake value [SUV] images. The purpose of this study was to systematically compare metabolic tumor volume assessments derived from SUV and Patlak images using a variety of (semi-)automatic tumor delineation methods in order to identify methods that can be used reliably on (whole body) SUV images.

**Methods:**

Dynamic [^18^F]-fluoro-2-deoxy-D-glucose [FDG] PET data from 10 lung and 8 gastrointestinal cancer patients were analyzed retrospectively. Metabolic tumor volumes were derived from both Patlak and SUV images using five different types of tumor delineation methods, based on various thresholds or on a gradient.

**Results:**

In general, most tumor delineation methods provided more outliers when metabolic volumes were derived from SUV images rather than Patlak images. Only gradient-based methods showed more outliers for Patlak-based tumor delineation. Median measured metabolic volumes derived from SUV images were larger than those derived from Patlak images (up to 59% difference) when using a fixed percentage threshold method. Tumor volumes agreed reasonably well (< 26% difference) when applying methods that take local signal-to-background ratio [SBR] into account.

**Conclusion:**

Large differences may exist in metabolic volumes derived from static and dynamic FDG image data. These differences depend strongly on the delineation method used. Delineation methods that correct for local SBR provide the most consistent results between SUV and Patlak images.

## Background

Positron-emission tomography [PET] may be used to delineate the biological target volume for both radiotherapy and response monitoring purposes [1-4]. The most widely used PET tracer, [^18^F]-fluoro-2-deoxy-D-glucose [FDG], might improve accuracy of tumor volume definition for radiotherapy by identifying areas within the tumor that are more metabolically active [5]. Tumor volumes can be delineated on either images of glucose metabolic rate or standardized uptake value [SUV] images [6]. SUV is most commonly used for (semi-)quantification of whole-body FDG PET studies and only requires a static scan. Images of glucose metabolic rate can be generated from dynamic scans using a measured or image-derived arterial input function together with Patlak graphical analysis. It is well known that Patlak analysis is quantitatively more accurate than SUV analysis. Patlak analysis, however, requires a dynamic scan and limits data acquisition to a single bed position with an axial coverage of < 20 cm.

As shown previously [6], metabolic volumes, defined using a 50% isocontour method, were smaller when defined on Patlak images than when defined on SUV images. To date, however, no systematic comparison has been performed in which various existing (semi-)automatic tumor delineation methods have been applied to both SUV and Patlak images.

Recently, a number of different (semi-)automatic tumor delineation methods have been validated for SUV images using both simulations [7,8] and lung tumor FDG scans [9,10]. Most methods showed good performance as measured maximum diameters derived from these methods corresponded well with pathological measurements [11]. As most of tumor delineation methods that correct for local background are less sensitive to changes in local contrast, these methods might show better correspondence between measured tumor volumes derived from either Patlak or SUV images. Therefore, the purpose of this study was to systematically compare measured metabolic tumor volumes derived from SUV and Patlak images using a variety of (semi-)automatic tumor delineation methods.

## Materials and methods

### Patient data

Dynamic FDG PET scans from 10 non-small cell lung cancer [NSCLC] (stages IIIB to IV) patients [12] and 8 gastrointestinal [GI] (colorectal carcinoma) cancer patients [13] were included retrospectively. All scans had been acquired prior to therapy. All patients had given written informed consent, and both studies had been approved by the Medical Ethics Review Committee of the VU University Medical Center.

For NSCLC patients (three females, seven males; weight 76 ± 10 kg, range 56 to 94 kg), blood glucose levels were within the normal range (mean 5.5 ± 0.6 mmol·L^-1^, range 4.4 to 7.0 mmol·L^-1^). The same was true for blood glucose levels (mean 5.6 ± 0.8 mmol·L^-1^, range 3.9 to 7.0 mmol·L^-1^) of patients with advanced GI malignancies (one female, seven males; weight 85 ± 15 kg, range 60 to 110 kg).

### PET scanning protocol

All patients fasted for at least 6 h before scanning. Patients were prepared in accordance with recently published guidelines for quantitative PET studies [14]. They were scanned in a supine position and received an intravenous catheter for tracer administration. During dynamic scanning, blood samples for determining plasma glucose levels were collected at fixed times (i.e., at 35, 45, 55 min post injection). All dynamic scans were performed using an ECAT EXACT HR+ scanner (Siemens/CTI, Knoxville, TN, USA) [15], having a 15.5-cm axial field of view. Each scan session started with a 10-min transmission scan using three retractable rotating ^68^Ge line sources. After completion of the transmission scan, a bolus of FDG was administrated intravenously (388 ± 71 and 459 ± 97 MBq for NSCLC and GI cancer, respectively), at the same time starting a dynamic emission scan in a 2-D acquisition mode. Each dynamic scan consisted of 40 frames with the following lengths, 1 × 30, 6 × 5, 6 × 10, 3 × 20, 5 × 30, 5 × 60, 8 × 150, 6 × 300 s. In addition, a static scan was created by summing the sinograms of the last three frames (i.e., 45 to 60 min post injection).

All data were normalized and corrected for attenuation, random coincidences, scatter radiation, dead time, and decay. Reconstructions were performed using normalization and attenuation-weighted ordered subsets expectation maximization [OSEM] with 2 iterations and 16 subsets, followed by post-smoothing using a 0.5 Hanning filter. This resulted in an image resolution of approximately 6.5 mm full width at half maximum. An image matrix size of 256 × 256 × 63 was used, corresponding to a pixel size of 2.57 × 2.57 × 2.43 mm^3^.

After reconstruction, the summed image (45 to 60 min post injection) was used to generate a SUV image by normalizing local tissue concentrations to injected dose and body weight. In addition, Patlak analysis, a kinetic linearized model [16] for irreversible tracer uptake, was applied to the interval 10 to 60 min post injection to generate an image of net influx rate [*K*_i_] of FDG, which is proportional to the metabolic rate of glucose. Image-derived input functions [IDIF] were used as plasma input curves and obtained as described by Cheebsumon et al. [17]. In short, 3-D volumes of interest [VOI] were drawn manually in three vascular structures (i.e., the left ventricle, aortic arch, and ascending aorta) using an early frame that highlights the blood pool [18]. These VOI were then projected onto all frames yielding arterial whole blood time activity curves (i.e., IDIF). The average input curves from VOI defined in the three vascular structures were used as an input function during Patlak analysis.

### Data analysis

For the 10 NSCLC patients, VOI were defined for 54 lesions that were all located in the lung. For the 8 GI cancer patients, VOI were defined for 37 lesions that were located in the liver (*n *= 23), lung (*n *= 12), or colon (*n *= 2). All lesions that could be identified by an expert physician were included in this study. Metabolic tumor volumes were obtained using the following five different types of (semi-)automatic VOI methods:

*Fixed threshold of 50% and 70% (VOI^50^, VOI^70^)*. In this method, a fixed threshold (i.e., 50% or 70%) of the maximum voxel value within a tumor is used to delineate the tumor [19].

*Adaptive threshold of 41%, 50%, and 70% (VOI^A41^, VOI^A50^, VOI^A70^)*. This is similar to the fixed threshold method, except that it adapts the threshold relative to the local average background, thereby correcting for the contrast between the tumor and local background [19].

*Contrast-oriented method (VOI^Schaefer^)*. This method uses the average of SUV within a 70% threshold of SUV_max _isocontour (meanSUV_70%_) and background activity for various sphere sizes. Regression coefficients are calculated, which represent the relationship between the optimal threshold and image contrast for various sphere sizes [3]. This threshold equation is given by:

$$\text{Threshol}{\text{d}}_{\text{optimal}}=A\times \text{meanSU}{\text{V}}_{70\%}+B\times \text{Background,}$$

where *A *and *B *are fitted using phantom studies [3]. When applied to Patlak images, *K*_i _rather than SUV is used. In general, different values are applied for sphere diameters smaller and larger than 3 cm in diameter. In the present study, this method was recalibrated, i.e., specific *A *and *B *values for the image characteristics used were determined.

*Background-subtracted relative-threshold level [RTL] method (VOI^RTL^)*. This method is an iterative method that is based on a convolution of the point-spread function, which takes into account differences between various sphere sizes and the scanner resolution [4].

*Gradient-based watershed segmentation method*. This method uses two steps before calculating the VOI [2]. First, a gradient image is calculated on which a 'seed' is placed in the tumor (tumor basin) and another in the background (background basin). Next, a watershed [WT] algorithm is used to grow both seeds in the gradient basins, thereby creating boundaries on the gradient edges. In the present study, two different types of gradient basins were used. In the first approach [Grad^WT1^], all voxels on the edge between the tumor and background are assigned to the tumor [8,10]. In the second approach [Grad^WT2^], an upsampled image is used to ensure less effects of sampling. In addition, a voxel on the edge between the tumor and background is allocated to either the tumor or background based on the smallest difference with that voxel value.

To reduce sensitivity to noise, for all methods, the maximum voxel value was obtained using a cross-shaped pattern. This method searches for the region with the (local) average maximum intensity based on the average of seven neighboring voxels, which was then used as a maximum or 'peak' value. The tumor-to-background ratio was calculated by dividing this maximum value by the background value surrounding the tumor.

### Statistical analyses

Both metabolic volumes and differences in measured volumes derived from two image types are reported. The percentage volume difference was defined as $\left(\frac{\text{Volum}{\text{e}}_{\text{SUV}}}{\text{Volum}{\text{e}}_{\text{Patlak}}}\text{ - }1\right)\times 100\%$. Note that this value can be negative, indicating an underestimation of the SUV-derived metabolic volume compared with the Patlak-derived volume. In addition, for each delineation method, mean, median, first quartile, third quartile, minimum, and maximum values, including statistical outliers, are reported in box plots. Moreover, visual outliers were identified as VOIs that showed unrealistically large or small volumes when compared visually with the tumor. These outliers were not included in the statistical analysis when calculating *p *values. A two-tailed Wilcoxon signed-rank test was used to indicate statistically significant differences between measured volumes derived from SUV and Patlak images, where *p *values less than 0.05 were considered to be significant.

## Results

Table 1 shows the number of visual outliers (i.e., those cases where there is an obvious mismatch between derived VOI and tumor boundaries) for all methods applied to both Patlak and SUV images, specifying results for NSCLC and GI cancer separately. In general, most tumor delineation methods provided more outliers when metabolic volumes were derived from SUV images rather than Patlak images. Only gradient-based methods showed more outliers for Patlak-based tumor delineation. VOI^70 ^and VOI^A70 ^provided no outliers for either image or cancer type.

**Table 1:** Number of visual outliers in SUV- and Patlak-derived measured metabolic volumes for both cancer

Delineation method	NSCLC		GI cancer	
	SUV image	Patlak image	SUV image	Patlak image
VOI^50^	9	-	5	3
VOI^70^	-	-	-	-
VOI^A41^	4	1	5	3
VOI^A50^	-	-	-	1
VOI^A70^	-	-	-	-
VOI^RTL^	-	1	3	-
VOI^Schaefer^	2	2	3	2
Grad^WT1^	-	3	13	15
Grad^WT2^	2	5	5	5

NSCLC, non-small cell lung cancer; GI, gatrointestinal; SUV, standardized uptake value; VOI, volumes of interest; Grad^WT1^, gradient-based watershed first approach; Grad^WT2^, gradient-based watershed second approach.

**Figure 1:**
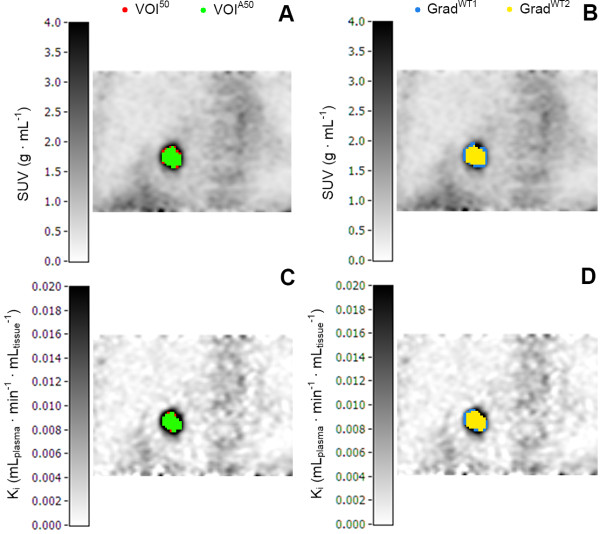
**Coronal images of the measured tumor volumes**. Coronal images of the measured tumor volumes derived from SUV and Patlak images of one patient with NSCLC, obtained using four different tumor delineation methods (i.e., VOI^50^, VOI^A50^, Grad^WT1^, and Grad^WT2^).

**Figure 2:**
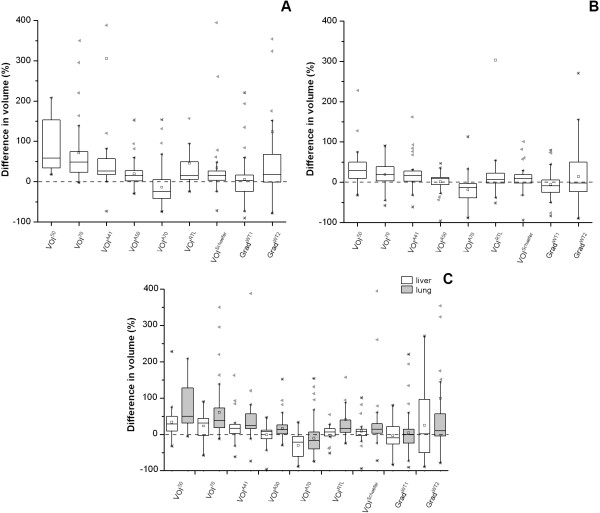
**Box-and-whisker plots of the percentage differences between measured volumes derived from SUV and Patlak images**. Box-and-whisker plots of the percentage differences between measured volumes derived from SUV and Patlak images for different tumor delineation methods in (**A**) NSCLC and (**B**) GI cancer, and (**C**) the pooled data from both studies specified per location, i.e., the liver and the lung. The median is the horizontal line between the lower (first) and upper (third) quartiles. Empty square represents the average value, cross, the minimum and maximum values, and filled left-pointing pointer, the number of statistical outliers. The percentage difference was defined as $\left(\frac{\text{Volum}{\text{e}}_{\text{SUV}}}{\text{Volum}{\text{e}}_{\text{Patlak}}}\text{ - }1\right)\times 100\%$.

**Figure 3:**
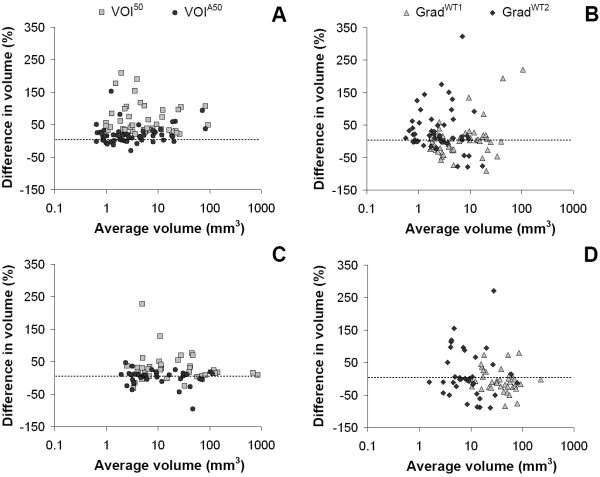
**Percentage difference in measured volumes compared to the measured volume**. Percentage difference in measured volumes compared to the measured volume derived from SUV and Patlak images for various delineation methods applied to (**A**, **B**) NSCLC and (**C**, **D**) GI cancer. Note that some data points (for VOI^50 ^and Grad^WT2^) fall outside the range of the figure. The percentage difference was defined as $\left(\frac{\text{Volum}{\text{e}}_{\text{SUV}}}{\text{Volum}{\text{e}}_{\text{Patlak}}}\text{ - }1\right)\times 100\%$.

**Figure 4:**
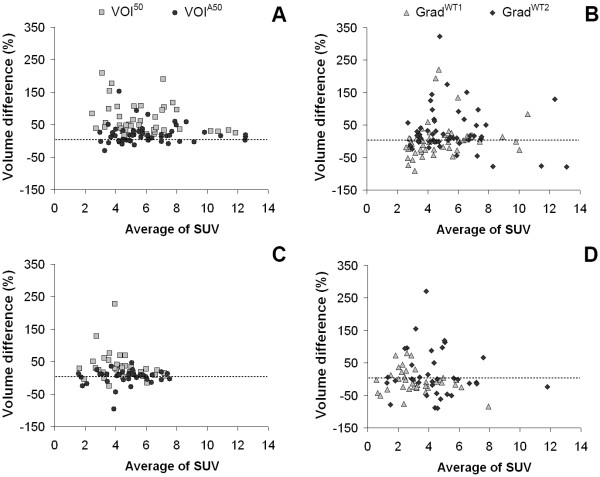
**Percentage difference in measured volumes compared to SUV**. Percentage difference in measured volumes compared to SUV derived from SUV and Patlak images for various delineation methods applied to (**A**, **B**) NSCLC and (**C**, **D**) GI cancer. Note that some data points (for VOI^50 ^and Grad^WT2^) fall outside the range of the figure. The percentage difference was defined as $\left(\frac{\text{Volum}{\text{e}}_{\text{SUV}}}{\text{Volum}{\text{e}}_{\text{Patlak}}}\text{ - }1\right)\times 100\%$.

In general, measured tumor volumes derived from SUV images were larger than those derived from Patlak images. Example images of the measured tumor volumes derived from SUV and Patlak images are shown in Figure 1. Exceptions were VOI^A70 ^for both types of cancer and the two gradient-based methods for GI cancer (Tables 2 and 3). Large differences (up to 58.7% and 28.1% for NSCLC and GI cancer, respectively) in measured metabolic volume based on the two image types were observed for the various delineation methods (Figure 2A). In the case of NSCLC, the median difference in volume was higher for fixed threshold methods than for adaptive, contrast-oriented, or gradient-based methods. This is further illustrated by Figure 3A, where VOI^A50 ^(i.e., with background correction) shows better correspondence between SUV- and Patlak-based volumes than VOI^50 ^(i.e., without background correction). Only Grad^WT1 ^provided no significant difference (*p *> 0.05) in the metabolic volume derived from SUV and Patlak images, but this may be due to the large spread in differences (Figure 3B). Similar results were found when these differences in volume were compared to SUV (or *K*_i _values, Figures 4A, B). In general, we observed that smaller lesions also had the lowest SUV. Consequently, the largest volume differences between SUV and Patlak image-based tumor delineations were seen for lesions having a low SUV and a small metabolic volume.

**Table 2:** Mean, median, minimum, and maximum values of metabolic volumes and their median differences for NSCLC

Delineation method	Volume obtained from SUV image (mL)				Volume obtained from Patlak image (mL)				% Median difference^a^	*P *value	*P *value^b^
	Mean	Median	Min	Max	Mean	Median	Min	Max			
VOI^50^	84.2	6.9	1.2	950.2	7.7	2.7	0.7	74.4	58.7	< 0.001	< 0.001
VOI^70^	4.9	1.5	0.5	50.2	2.5	1.1	0.3	23.7	48.0	< 0.001	< 0.001
VOI^A41^	22.7	5.2	1.2	294.4	10.9	3.5	0.9	95.2	25.8	< 0.001	< 0.001
VOI^A50^	8.8	2.4	0.6	97.0	6.6	2.4	0.5	70.6	15.0	< 0.001	< 0.001
VOI^A70^	2.1	0.5	0.1	23.4	1.8	0.8	0.2	19.9	-25.0	0.044	0.044
VOI^RTL^	10.4	3.6	0.4	101.9	7.7	3.1	0.3	73.1	15.6	< 0.001	< 0.001
VOI^Schaefer^	17.2	5.1	1.0	125.7	13.8	4.2	0.9	104.2	14.3	< 0.001	< 0.001
Grad^WT1^	12.3	3.9	1.4	163.2	10.4	5.4	1.6	50.9	1.9	0.725	0.324
Grad^WT2^	5.8	2.7	0.6	63.2	3.7	2.1	0.5	28.2	18.2	< 0.001	0.001

^a^The percentage difference was defined as $\left(\frac{\text{Volum}{\text{e}}_{\text{SUV}}}{\text{Volum}{\text{e}}_{\text{Patlak}}}\text{ - }1\right)\times 100\%$. The average tumor-to-background ratio was 5.3 (range 2.7 to 12.7) and 18.8 (range 6.1 to 81.0) when derived from SUV and Patlak images, respectively. ^b^Without visual outliers. SUV, standardized uptake value; min, minimum; max, maximum; VOI, volumes of interest; Grad^WT1^, gradient-based watershed first approach; Grad^WT2^, gradient-based watershed second approach.

**Table 3:** Mean, median, minimum, and maximum values of metabolic volumes and median differences for GI cancer

Delineation method	Volume obtained from SUV image (mL)				Volume obtained from Patlak image (mL)				% Median difference^a^	*P *value	*P *value^b^
	Mean	Median	Min	Max	Mean	Median	Min	Max			
VOI^50^	190.4	15.4	2.7	2297.8	65.2	10.1	2.1	822.9	28.1	< 0.001	< 0.001
VOI^70^	10.5	3.9	1.3	57.6	8.8	3.5	1.0	45.2	18.5	< 0.001	< 0.001
VOI^A41^	195.4	28.6	3.3	2402.5	86.1	11.9	2.5	1257.6	16.5	< 0.001	< 0.001
VOI^A50^	20.3	6.0	2.1	121.2	22.2	6.8	1.9	107.3	8.1	0.364	0.215
VOI^A70^	5.1	1.7	0.7	38.8	6.57	2.38	0.51	34.26	-13.3	0.001	0.001
VOI^RTL^	33.3	7.2	0.3	538.2	17.78	6.75	0.26	111.14	7.5	0.040	0.042
VOI^Schaefer^	158.0	14.7	3.4	2212.0	48.1	13.0	2.5	564.6	8.3	0.003	0.004
Grad^WT1^	43.5	32.8	9.1	223.5	51.2	43.2	10.2	229.4	-9.1	0.025	0.085
Grad^WT2^	12.4	6.8	1.5	74.1	14.2	8.6	1.6	85.3	-2.1	0.625	1.000

^a^The percentage difference was defined as $\left(\frac{\text{Volum}{\text{e}}_{\text{SUV}}}{\text{Volum}{\text{e}}_{\text{Patlak}}}\text{ - }1\right)\times 100\%$. The average tumor-to-background ratio was 7.4 (range 2.4 to 31.6) and 16.0 (range 3.0 to 32.0) when derived from SUV and Patlak images, respectively. ^b^Without visual outliers. SUV, standardized uptake value; min, minimum; max, maximum; VOI, volumes of interest; Grad^WT1^, gradient-based watershed first approach; Grad^WT2^, gradient-based watershed second approach.

Similar trends were observed for GI cancer (Figures 2B and 3C, D). Here, Grad^WT1 ^(only after removal of visual outliers), Grad^WT2^, and VOI^A50 ^provided no significant differences (*p *> 0.05) between measured volumes derived from both image types. In addition, similar trends were observed when data from both studies were pooled and presented separately for the specific locations of the tumors (i.e., the liver or the lung, Figure 2C).

## Discussion

The main use of FDG is measurement of glucose metabolism. However, FDG PET can also be used to measure the volume with increased metabolism. In a previous report [6], it was shown that tumor delineation using Patlak-derived glucose metabolism images provided smaller volumes and sharper borders than when SUV images were used. This was due to a higher local contrast in Patlak images than in SUV images. Patlak analysis, however, may not always be feasible or optimal because it requires (measured) arterial input data and a dynamic scan, which limits coverage to a single bed position. Therefore, in clinical practice, a static whole-body scan (covering the whole body) might be preferred, in which case data can only be analyzed using a SUV approach. It is well known, however, that SUV may be affected by technical, biological, and physical factors [20] that could hamper tumor delineation using this image type.

In agreement with Visser et al. [6], the present study showed (for two types of cancer) that when a fixed percentage threshold method (i.e., VOI^50^) was used, significantly larger metabolic volumes were obtained from SUV images than from Patlak images. However, these differences reduced when using methods that correct for local background and/or contrast, and in the case of gradient-based methods (Figure 2, Tables 2 and 3). This confirms that SUV-based tumor delineation is sensitive to signal-to-background ratios. Differences in Patlak- and SUV-derived volumes were larger for NSCLC than for GI cancer, especially in the case of methods that use a fixed percentage threshold without background correction. As local (image) contrast for GI cancer was larger than that for NSCLC (average tumor-to-background ratios 7.4 and 5.3, respectively), this further illustrates the sensitivity to signal-to-background ratio.

Some tumor delineation methods (i.e., VOI^50^, VOI^A41^, and Grad^WT1^) provided visually, unrealistically large tumor volumes (in up to 41% of cases) when applied to SUV images, while VOI^Schaefer ^did the same (in up to 8% of cases) for both image types (Table 1). In contrast, Grad^WT2 ^provided many unrealistically small tumor volumes (in up to 24% of cases) for both image types. This suggests that these methods should be applied carefully and that their performance should be supervised.

Two different implementations of gradient-based methods were evaluated in the present study. In a previous NSCLC study [11], tumor diameters obtained using Grad^WT2 ^corresponded better to pathology than those obtained using Grad^WT1^. As shown in Figure 3B, the present study also showed that measured volumes obtained from Grad^WT2 ^were smaller than those from Grad^WT1^. However, Grad^WT1 ^showed better correspondence between SUV- and Patlak-derived volumes than Grad^WT2 ^(1.9% and 18.2%, respectively). In contrast, for GI cancer, Grad^WT2 ^showed better correspondence between SUV- and Patlak-derived volumes than Grad^WT1 ^(-2.1% and -9.1%, respectively). This suggests that the performance of gradient-based methods may also depend on signal-to-background ratios.

Differences between metabolic volumes obtained from SUV and Patlak images reduced when signal-to-background-corrected delineation methods are used. This finding is in line with previous studies reporting on test-retest variability using various tumor delineation methods [10,21] that confirmed that VOI^A50 ^seems to be a good possible candidate for response monitoring purposes. However, gradient-based methods have been shown to be good candidates for radiotherapy purposes [10,22]. Therefore, either signal-to-background-corrected or gradient-based methods may be good candidates for response assessments and radiotherapy purposes.

## Limitations

A limitation of this study is the lack of an independent reference standard to define tumor volumes, and consequently, in this paper, we could only study differences in (semi-)automatic tumor delineation method performance when applied onto Patlak versus SUV images. However, the accuracy and precision of several (semi-)automatic tumor delineation methods have been studied previously using simulations [8] and clinical test-retest data [10]. Both articles showed that performance of tumor delineation methods are affected by several factors, such as scanner type, radiotracer, image noise, and tumor characteristics. It is generally accepted that pathology is the gold standard. Therefore, studies are needed and are currently performed that compare the tumor volumes obtained using (semi-)automatic delineation methods with pathology [11].

Although the Patlak analysis was performed on OSEM-reconstructed images in order to reduce the levels of noise, Patlak images still showed a small fraction (< 1%) of voxels that had a negative slope, exclusively seen in non-tumor tissue locations. Correlation-coefficient filtered parametric imaging, as proposed by Zasadny and Wahl [23], could potentially enhance the quality of the Patlak images and could be further investigated to improve the accuracy of automated tumor delineation. Despite the lack of using such a denoising method, our results were in line with those published by Visser et al. [6], and we could identify that tumor delineation methods that correct for local signal-to-background contrast or use gradients showed a better agreement in tumor volume assessment between Patlak and SUV images than those tumor delineation methods that did not.

## Conclusion

Large differences may exist in metabolic volumes derived from static (SUV) and dynamic (Patlak) FDG image data. These differences depend strongly on the delineation method used. (Semi-)automatic tumor delineation methods that correct for local signal-to-background contrast or use gradients provide the most consistent results between SUV and Patlak images.

## Competing interests

The authors declare that they have no competing interests.

## Authors' contributions

PC performed the data analysis and data interpretation and was the main author of the manuscript. FHPvV performed the data interpretation, implemented some of the tumor delineation methods, and assisted in drafting the manuscript. MY implemented some of the tools to perform tumor delineations and critically reviewed the manuscript. CJH performed part of the data acquisition and critically reviewed the manuscript. LMV provided the PET image data and reviewed the manuscript. WH provided/collected the PET image data and reviewed the manuscript. OSH reviewed the manuscript and approved its final content. AAL reviewed the manuscript, contributed to the intellectual content (supervision), and approved the final content of the manuscript. RB performed the study design, implemented some of the tumor delineation methods, supervised the project, and reviewed and approved the final content of the manuscript. All authors reviewed the collected data and interpretation, provided feedback for further research during the study, and approved the final submitted version of this manuscript.
